# A data-driven model of disability progression in progressive multiple sclerosis

**DOI:** 10.1093/braincomms/fcae434

**Published:** 2024-12-03

**Authors:** Sara Garbarino, Carmen Tur, Marco Lorenzi, Matteo Pardini, Michele Piana, Antonio Uccelli, Douglas L Arnold, Bruce A C Cree, Maria Pia Sormani, Francesca Bovis

**Affiliations:** Life Science Computational laboratory, IRCCS Ospedale Policlinico San Martino, 16132 Genoa, Italy; MIDA, Dipartimento di Matematica, Università di Genova, 16146 Genoa, Italy; Multiple Sclerosis Centre of Catalonia (Cemcat), Department of Neurology, Hospital Universitari Vall d’Hebron, Universitat Autònoma de Barcelona, 08035 Barcelona, Spain; Universitè Côte d’Azur, Inria, Epione Research Project, 06902 Sophia Antipolis, France; Department of Neuroscience, Rehabilitation, Ophthalmology, Genetics, Maternal and Child Health, Università di Genova, 16132 Genoa, Italy; IRCCS Ospedale Policlinico San Martino, 16132 Genoa, Italy; Life Science Computational laboratory, IRCCS Ospedale Policlinico San Martino, 16132 Genoa, Italy; MIDA, Dipartimento di Matematica, Università di Genova, 16146 Genoa, Italy; Department of Neuroscience, Rehabilitation, Ophthalmology, Genetics, Maternal and Child Health, Università di Genova, 16132 Genoa, Italy; IRCCS Ospedale Policlinico San Martino, 16132 Genoa, Italy; McGill University, H3A0G4 Montréal, Canada; UCSF Weill Institute for Neurosciences, Department of Neurology, University of California San Francisco, 94143 San Francisco, USA; IRCCS Ospedale Policlinico San Martino, 16132 Genoa, Italy; Department of Health Sciences (DISSAL), Università di Genova, 16132 Genoa, Italy; Department of Health Sciences (DISSAL), Università di Genova, 16132 Genoa, Italy

**Keywords:** primary progressive multiple sclerosis, Bayesian learning, data-driven disease progression modelling, PPMS sub-groups, multimodal data

## Abstract

This study applies the Gaussian process progression model, a Bayesian data-driven disease progression model, to analyse the evolution of primary progressive multiple sclerosis. Utilizing data from 1521 primary progressive multiple sclerosis participants collected within the International Progressive Multiple Sclerosis Alliance Project, the analysis includes 18 581 longitudinal time-points (average follow-up time: 28.2 months) of disability assessments including the expanded disability status scale, symbol digit modalities, timed 25-foot-walk, 9-hole-peg test and of MRI metrics such as T1 and T2 lesion volume and normalized brain volume. From these data, Gaussian process progression model infers a data-driven description of the progression common to all individuals, alongside scores measuring the individual progression rates relative to the population, spanning ∼50 years of disease duration. Along this timeline, Gaussian process progression model identifies an initial steep worsening of the expanded disability status scale that stabilizes after ∼30 years of disease duration, suggesting its diminished utility in monitoring disease progression beyond this time. Conversely, it underscores the slower evolution of normalized brain volume across the disease duration. The individual progression rates estimated by Gaussian process progression model can be used to identify three distinct sub-groups within the primary progressive multiple sclerosis population: a normative group (76% of the population) and two ‘outlier’ sub-groups displaying either accelerated (13% of the population) or decelerated (11%) progression compared to the normative one. Notably, fast progressors exhibit older age at symptom onset (38.5 versus 35.0, *P* < 0.0001), a higher prevalence of males (61.1% versus 48.5%, *P* = 0.013) and a higher lesion volumes both in T1 (4.1 versus 0.6, *P* < 0.0001) and T2 (16.5 versus 7.9, *P* < 0.0001) compared to slow progressors. Prognostically, fast progressors demonstrate a significantly worse prognosis, with double the risk of experiencing a 3-month confirmed disease progression on expanded disability status scale compared to the normative population according to Cox proportional hazard modelling (HR = 2.09, 95% CI: 1.66–2.62, *P* < 0.0001) and a shorter median time from the onset of disease symptoms to reaching a confirmed expanded disability status scale 6 (95% CI: 5.83–7.68 years, *P* < 0.0001). External validation on a test set comprising 227 primary progressive multiple sclerosis participants from the SPI2 trial produced consistent results, with slow progressors exhibiting a reduced risk of experiencing 3-month confirmed disease progression determined through expanded disability status scale (HR = 0.21), while fast progressors facing an increased risk (HR = 1.45). This study contributes to our understanding of disability accrual in primary progressive multiple sclerosis, integrating diverse disability assessments and MRI measurements. Moreover, the identification of distinct sub-groups underscores the heterogeneity in progression rates among patients, offering invaluable insights for patient stratification and monitoring in clinical trials, potentially facilitating more targeted and personalized interventions.

## Teaser

Garbarino *et al*. analysed a large cohort of progressive multiple sclerosis patients and identified patterns of long-term disability and cognitive decline. The findings suggest significant heterogeneity in disease trajectories, identifying two groups with either accelerated or decelerated progression compared to the normative population, highlighting potential markers for disease progression.

## Graphical Abstract

**Graphical Abstract:**
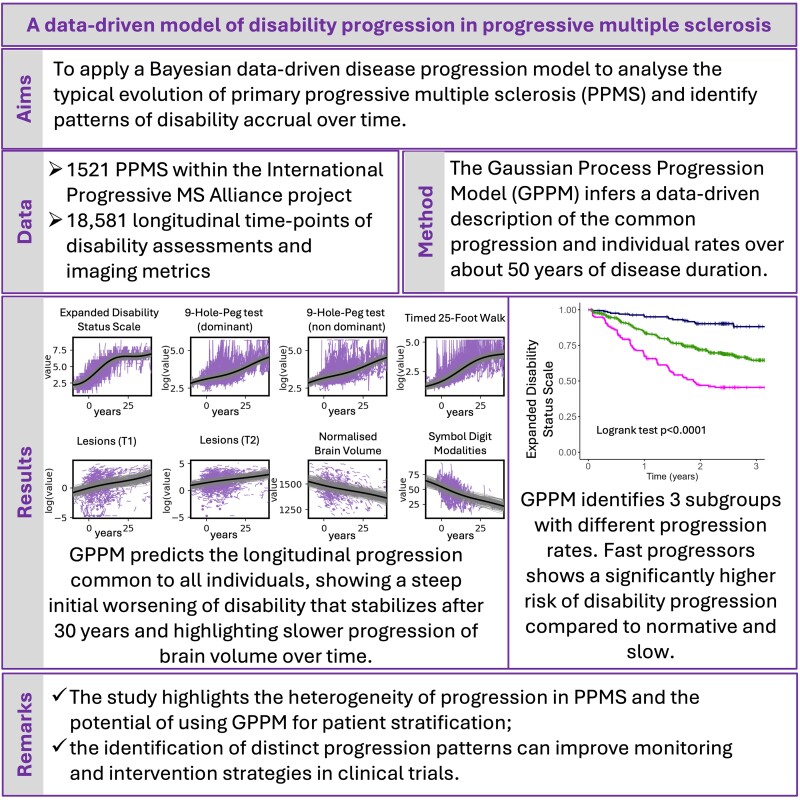

See P. Oxtoby and Barkhof Leckey and Zetterberg (https://doi.org/10.1093/braincomms/fcae474) for a scientific commentary on this article.

## Introduction

Despite significant advancements in understanding the underlying mechanisms of multiple sclerosis (MS) and the availability of treatments to prevent relapses, there remains an unmet need to halt and reverse disability accrual. Accumulated evidence suggests that disability accrual in MS is not driven by a single, uniform disease mechanism but rather by a combination of several mechanisms that vary among patients and even within individual patients over time.^1^ Quantifying disability accrual presents a notable challenge, particularly in the context of progressive multiple sclerosis (PMS) patients. The heterogeneous nature of disability manifestations,^2-10^ the prolonged progression spanning years and the limitations inherent in the measures employed to quantify progression collectively pose a significant challenge in the analysis of PMS data. In the face of the persistent unknowns surrounding disease progression, elucidating the timeline of disability accrual holds the promise of unveiling crucial insights into the underlying mechanisms of disease progression, thereby facilitating patient stratification and monitoring in clinical trials.

Data-driven disease progression models^11^ are a set of computational tools specifically designed to quantify the long-term timeline of progression from short-term multimodal clinical data. These models can reveal disability patterns in a wide variety of sporadic and familial neurological diseases including Alzheimer’s disease,^12-21^ Huntington’s disease,^22,23^ Parkinson’s disease,^24^ frontotemporal dementia^25^ and progressive supranuclear palsy.^26,27^ Two recent studies involving PMS patients^28,29^ determined the most likely sequence in which brain regions become atrophic; however, they analysed only MRI structural metrics and did not include features derived from other modalities, such as disability or cognition measures, or lesion volumes. In a more recent study,^30^ the authors considered a broader set of structural, functional and cognitive outcomes, on a limited dataset of relapsing-onset MS subjects.

This study aims to elucidate the typical pattern of disability accrual in PMS by analysing a large dataset of randomized clinical trial data encompassing longitudinal multimodal disability assessments (walking ability, upper limb function and cognitive function) and MRI measurements (lesion and brain volume) collected within an International Progressive MS Alliance project. We employ Gaussian process progression model (GPPM), a probabilistic data-driven disease progression model, previously used to uncover disease progression in Alzheimer’s Disease^17-19^ as well as longitudinal evolution of ageing.^20^ The aim of this analysis is to better characterize the typical progression of primary progressive MS (PPMS) and identify potential sub-groups of outliers with respect to such progression.

## Materials and methods

### Training dataset

Three randomized clinical trials of participants with PPMS collected within the International Progressive MS Alliance project (IPMSA, award reference number PA-1603–08175) were included in this analysis: ARPEGGIO, OLYMPUS and ORATORIO (ClinicalTrials.gov numbers, NCT02284568, NCT00087529 and NCT01194570 respectively). The study design and inclusion/exclusion criteria of the trials were previously described.^31-33^ The ethics committees and institutional review boards of all participating centres approved the study protocols. All participants provided written informed consent.

Longitudinal metrics were analysed and included the expanded disability status scale (EDSS), the Timed 25-Foot Walk test (T25FW), the 9-Hole Peg Test (9HPT) (dominant and non-dominant hand), the Symbol Digit Modalities test (SDMT, a cognitive test of attention), and MRI markers including T1 and T2 lesion volume and normalized brain volume (NBV). Participants with missing disease duration were excluded from the analysis (22 participants from ORATORIO trial and one each from the ARPEGGIO and OLYMPUS trials). The variables that were not normally distributed (lesion volumes, T25FW and 9HPT measures) were log-transformed for the analysis. Finally, all the measurements were converted into quantile scores (0–1 for normal to abnormal values), according to their distribution. Disease duration was collected as well, and is considered from the onset of symptoms (as reported by the participants).

### Test dataset

The PPMS participants (*N* = 227) from the SPI2 study (ClinicalTrials.gov number NCT02936037) were used as an independent external validation set.^34^ The same variables as the training set were collected, except for 9HPT measurements that were not assessed in this trial.

### Disease progression modelling using GPPM

GPPM, a data-driven Bayesian progression method, was used to model PPMS progression on the training dataset. GPPM is a mixed-effect model designed to handle repeated measures data by incorporating both fixed effects (e.g. the progression of the pathology common to all individuals) and random effects (e.g. individual-specific variability), as well as scores measuring the individual rates of progression, relative to the population.^17^ The model does so by assuming that measurements are taken at an unknown ‘disease-time’, represented by a hidden variable to be estimated for each individual, while fitting the set of group-wise trajectories across subjects. The resulting disease-time axis parametrizes the natural evolution of the disability common to all individuals, while the individual disease-time scores (also called ‘time reparameterization parameters’) are measures of individual progression severity with respect to the common disease-time.

Formally $y^{s}(t_{1}),\ldots ,y^{s}(t_{T^{s}})$ represent the longitudinal multimodal measurements associated with each individual *s* at corresponding study time points $(t_{1},\ldots ,t_{T^{s}}$). It is then possible to consider all measurements obtained at a particular visit of one individual *s* to occur at an ideal disease time $\tau_{\;j^{s}}$, where $j^{s}=1,\ldots ,T^{s}$ and the mapping from study time *t* to disease time *τ* is modelled by a subject-specific time reparametrization function, assumed to be a time-shift: $t_{\;j^{s}}=\;\tau_{\;j^{s}}+d^{s}$, for some parameters $d^{s}$. Under this assumption, the observations (for all clinical and MRI progression markers k=1,…,K) for subject *s* at a given time point *t* (indices omitted but implied) can be modelled as a random sample from the model:


$$y^{s}(t):\;=(y_{1}^{s}(t),\ldots ,y_{K}^{s}(t))=f(t)+\eta^{s}(t)+\varepsilon$$

Here, $f(t)=(\;f_{1}(t),\ldots ,f_{K}(t))$ is a Gaussian Process; $\eta^{s}(t)=(\eta_{1}^{s}(t),\ldots ,\eta_{K}^{s}(t))$ are individual Gaussian random effects and $\varepsilon =(\varepsilon_{1},\ldots \;\varepsilon_{K})$ is the measurement noise, assumed to be Gaussian heteroskedastic. The model was previously described in detail together with the optimization scheme to recover the probabilistic estimates of the parameters for the fixed effect, the random effect, and the individual time reparameterization parameters.^17,18,35^ Identifiability of the model is ensured by enforcing global monotonicity on the population-level trajectories. Also, as the covariance of the Gaussian Process can be specified in order to account for incomplete data, the model naturally accounts for missing information and no data imputation is needed. Explicit estimations of the predictive performance of the model in assessing the individual parameters with respect to follow-up assessments and missing values can be found in.^17^ Further, the Bayesian formalism naturally allows for probabilistic estimation of the disability trajectories and uncertainty quantification of the predicted individual time reparametrization parameters, without the need for cross-validation or bootstrapping. Finally, Gaussian processes naturally allow for probabilistic predictions of unseen individuals given the individual clinical and MRI progression marker measurements of the training sample.^17^

In this study, GPPM was run on the training set and used the observed participants’ disease duration (ODD) at visit as reference for input study time frame ***t***. Consequently, GPPM estimates the individual reparametrized disease durations (RDD) as measures of progression stages, representing a proxy for the disease time frame ***τ***. Because the model requires monotonicity constraints to fit population-level trajectories, monotonic non-increasing constraints were enforced on SDMT and NBV, and non-decreasing constraints on the six remaining clinical and MRI progression markers.

### Outliers identification using reparametrized disease duration

Following GPPM modelling on the training dataset, the individual time reparameterization parameters were examined, which measure the differences between the individual ODD and the model’s RDD. By doing so, one ‘normative’ sub-group was identified, in which the individuals had an estimated time reparameterization parameter, which was within 1 SD from the population level average parameter. Additionally, two ‘outlier groups’ with respect to the normative progression estimated by the model were identified, i.e. individuals with an estimated time reparameterization parameter, which was above or below 1 SD from the population level average parameter. Specifically, these three groups were denoted as:

1. Slow progressors (G1)—participants with an associated reparameterization parameter falling below 1 SD from the population-level average parameter.
2. Normative progressors (G2)—participants whose time reparameterization parameter is within ±1 SD of the population-level average parameter.
3. Fast progressors (G3)—participants with an associated reparameterization parameter exceeding 1 SD from the population-level average parameter.

### Statistical analysis

The demographic and clinical characteristics of the three populations defined by the GPPM were described using appropriate measures such as mean (standard deviation [SD]), median (interquartile range [IQR]), or frequencies (percentage), depending on the data distribution. These characteristics were compared among sub-groups by employing a χ^2^ test for categorical variables, and utilizing ANOVA or Kruskal–Wallis test, depending on the nature of the variables, for continuous measures.

To assess whether using the ODD alone was effective to identify the normative and outlier subpopulations, a ‘null model’ was implemented. This involved classifying three distinct subpopulations based solely on the ODD, where the average ODD for the entire population was computed along with its SD. Specifically, three ‘null’ sub-groups were defined (following what we did for G1, G2 and G3): a normative ‘null’ sub-group, in which the individuals had an ODD within 1 SD from the population-level average ODD; and two outliers ‘null’ groups, in which the individuals had an ODD above or below 1 SD from the population-level ODD. Demographic and clinical characteristics of the three ‘null’ populations and their 3-month confirmed disability progression (CDP) were then assessed and compared to what was obtained when looking at the three populations defined by the GPPM.

A Cox proportional hazards model was used to evaluate if there was a difference in the risk of 3-month CDP computed on EDSS, T25FW, SDMT and 9HPT (both dominant and non-dominant), among the three populations defined by the GPPM. CDP calculated on EDSS was defined as a 1-point increase in EDSS if the baseline score was 3.0–5.0 or a 0.5-point increase if the baseline score was 5.5–6.5, confirmed at a scheduled visit at least 3 months later. CDP computed on T25FW and 9HPT was defined as a worsening of at least 20% from baseline confirmed at a scheduled visit at least 3 months later. CDP computed on SDMT was defined as 6-month confirmed worsening of ≥3 points or 10% of the SDMT score. Age at symptoms onset, sex and treatment arm were included in the multivariable Cox regression model as covariates.

The median time and age at which an EDSS of 6 occurred were computed using the Kaplan–Meier approach. To compare the survival curves, the log-rank test was employed, and a two-sided *P*-value was reported without any correction for multiple comparisons.

## Results

A total of 1521 PPMS participants (18 581 time-points) (373 from ARPEGGIO, 438 from OLYMPUS and 710 from ORATORIO) were analysed, with complete or partial availability of the 8 disease evolution measures. 998/1521 (65.61%) participants were treated (ARPEGGIO 233/373 (62.47%); OLYMPUS: 292/438 (66.67%); ORATORIO: 473/710 (66.2%)). Demographic and clinical characteristics of the training cohort are described in Table 1. Mean follow-up duration of the pooled training dataset was 28.20 months (SD: 10.79 months), with a median frequency of EDSS assessments of 2.76 months (IQR: 2.66–2.79 months). Median frequency of 9HPT, T25FW and SDMT assessments was every 2.76 months (IQR: 2.70–2.79 months) for all three disease measures. MRI measurements have comparable median frequencies. SDMT values were collected solely in the ARPEGGIO trial, leading to a relatively small number of participants with this information available (*N* = 363). Similarly, the volume of lesions in T1 was not available for the OLYMPUS trial and was available for the ORATORIO trial and for 58 participants in the ARPEGGIO trial.

**Table 1:** Demographic and clinical characteristics of the training dataset

	Overall population	Slow progressors	Normative progressors	Fast progressors	*P*-value
*N*	1521	167	1161	193	
Age at symptoms onset (years), mean (SD)	38.94 (8.79)	34.99 (9.73)	39.58 (8.39)	38.51 (9.40)	<0.0001
Male sex, *n* (%)	781 (51.35)	81 (48.50)	582 (50.13)	118 (61.14)	0.013
Disease duration at baseline, median (IQR)	6.26 (3.73–9.79)	12.90 (9.23–18.03)	5.86 (3.46–8.57)	5.81 (3.30–9.34)	<0.0001
EDSS at baseline, median (IQR)	4.50 (3.50–6.00)	3.50 (3.00–4.50)	4.50 (3.50–5.50)	6.00 (6.00–6.50)	<0.0001
	*N* = 1500		*N* = 1142	*N* = 191	
T25FW at baseline, median (IQR)	7.60 (5.70–12.25)	5.97 (5.00–7.65)	7.27 (5.70–10.35)	20.20 (13.05–36.50)	<0.0001
	*N* = 1490	*N* = 166	*N* = 1136	*N* = 188	
9HPT (dominant) at baseline, median (IQR)	25.55 (21.70–32.35)	22.40 (19.75–26.35)	25.15 (21.60–30.75)	37.40 (29.02–54.80)	<0.0001
	*N* = 1486	*N* = 165	*N* = 1133	*N* = 188	
9HPT (non-dominant) at baseline, median (IQR)	26.95 (22.80–34.02)	23.62 (19.85–27.82)	26.55 (22.75–32.50)	39.10 (30.60–60.50)	<0.0001
	*N* = 1484	*N* = 164	*N* = 1133	*N* = 187	
SDMT at baseline, median (IQR)^a^	44.00 (34.00–51.00)	50.00 (37.00–58.00)	45.00 (34.50–51.00)	30.50 (21.50–37.00)	<0.0001
	*N* = 363	*N* = 39	*N* = 304	*N* = 20	
NBV (ml) at baseline, mean (SD)	1380.27 (157.42)	1330.14 (185.17)	1389.72 (154.42)	1368.84 (143.61)	0.0002
	*N* = 1128	*N* = 120	*N* = 840	*N* = 168	
T2 lesion volume (ml), median (IQR)	5.82 (2.05–13.60)	7.85 (2.36–13.54)	5.35 (1.98–12.79)	16.49 (6.43–39.78)	<0.0001
	*N* = 1203	*N* = 14	*N* = 1125	*N* = 64	
T1 lesion volume (ml), median (IQR)^b^	1.73 (0.45–5.82)	0.59 (0.21–3.58)	1.64 (0.46–5.15)	4.14 (0.82–12.24)	<0.0001
	*N* = 765	*N* = 55	*N* = 594	*N* = 116	
ARR during follow-up, mean (SD)	0.06 (0.45)	0.02 (0.10)	0.06 (0.50)	0.07 (0.33)	0.436
Relapse during follow-up, *n* (%)					0.708
0	1414 (92.97)	158 (94.61)	1080 (93.02)	176 (91.19)	
1	86 (5.65)	8 (4.79)	65 (5.60)	13 (6.74)	
>1	21 (1.38)	1 (0.60)	16 (1.38)	4 (2.07)	
Treated patients, *n* (%)	998 (65.61)	116 (69.46)	762 (65.63)	120 (62.18)	0.349
Follow-up (months), mean (SD)	28.20 (10.79)	28.57 (9.11)	28.03 (11.01)	28.92 (10.82)	0.488
Trial, *n* (%)					<0.0001
ARPEGGIO	373 (24.52)	39 (23.35)	314 (27.05)	20 (10.36)	
OLYMPUS	438 (28.80)	77 (46.11)	304 (26.18)	57 (29.53)	
ORATORIO	710 (46.68)	51 (30.54)	543 (46.77)	116 (60.10)	

SD, standard deviation; EDSS, expanded disability status scale; IQR, interquartile range; T25FW, Timed 25-Foot Walk test; 9HPT, 9-Hole Peg Test; SDMT, Symbol Digit Modalities test; NBV, normalized brain volume; ARR, annualized relapse rate.

^a^The SDMT data are available only in ARPEGGIO trial.

^b^Data are available for 58 patients in ARPEGGIO trial and is not available in OLYMPUS trial.

### Progression of primary progressive multiple sclerosis patients

**Figure 1:**
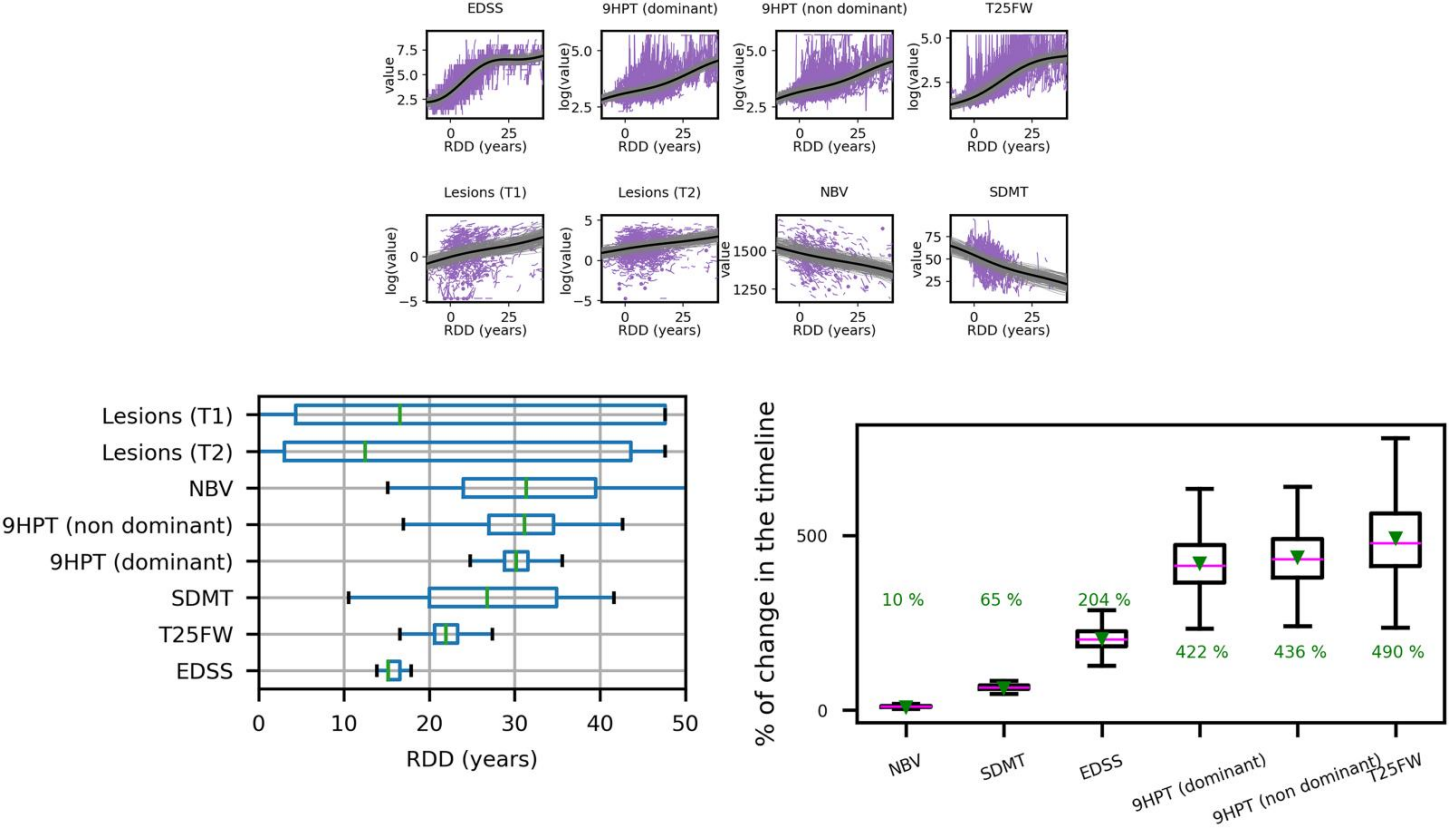
**Estimated progression on the training set.** Top: Panel with progression. On the *x*-axis, the RDD. The central solid lines represent the estimated average trajectory, while the ones around are 100 realizations from the average one. The individual measurements are displayed as two or more measurements connected with a segment (longitudinal data), or dots (cross-sectional data); bottom-left: temporal ordering in which the disability assessments and the MRI metrics reach their highest rate of change along the estimated progression; bottom-right: global percentage of change for PPMS along the entire estimated timeline. The mean is highlighted as a solid line while the median is shown as a green triangle. For all plots, *N* = 1500 subjects had EDSS measurements; *N* = 1486 had 9HPT (dominant); *N* = 1484 had 9HPT (non-dominant); *N* = 1490 had T25FW; *N* = 1203 had T1 lesion volumes; *N* = 1203 had T2 lesion volumes; *N* = 1128 had NBV; *N* = 363 had SDMT.

Figure 1 top shows the estimated clinical and MRI progression markers trajectories, over an estimated disease-time axis of roughly 50 years (48.7 years). It also shows individual measurements, shifted according to the estimated RDD. Indeed, it is characterized at the initial stages by a sharp increase for EDSS, which then reaches a plateau after about 20–30 years of disease duration. The other disability markers (T25FW and both dominant and non-dominant 9HPT) get steadily worse along progression, similarly to lesion volumes. These latter measures are however heterogeneously distributed in the population and with very large variability. The evolution is further characterized by a steady worsening of SDMT, starting at roughly 25 years of disease duration, followed by NBV loss. These measures are again quite variable in the population, likely due to the large number of missing data (see Table 1).

Similar conclusions can be drawn by looking at Fig. 1 bottom-left, which shows the temporal ordering in which the clinical and MRI progression markers reach their highest rate of change along the estimated timeline. Except for lesion volumes, whose variability is spread across the entire timeline, the model predicts a sequence disease progression characterized by initial changes of EDSS, followed by T25FW and SDMT. Changes in 9HPT dominant and non-dominant appear at a later stage—the latter one with higher associated uncertainty—and finally NBV loss. Supplementary Fig. S1 supports this results using an alternative discrete disease progression model,^11^ the z-score SuStain^13,36^ constrained to a single progression type. The estimated sequence broadly aligns with that derived from GPPM, with EDSS and disability-related features appearing early, while the most significant changes in NBV, SDMT, and lesion markers occur later in the progression, although some initial decline is also observed.

Figure 1 bottom-right shows the global percentages of change in the estimated timeline of 48.7 years for six out of the eight markers: EDSS, NBV, SDMT, T25FW and 9HPT (dominant and non-dominant).

### Clinical characteristics of the outlier groups with respect to the normative population

**Figure 2:**
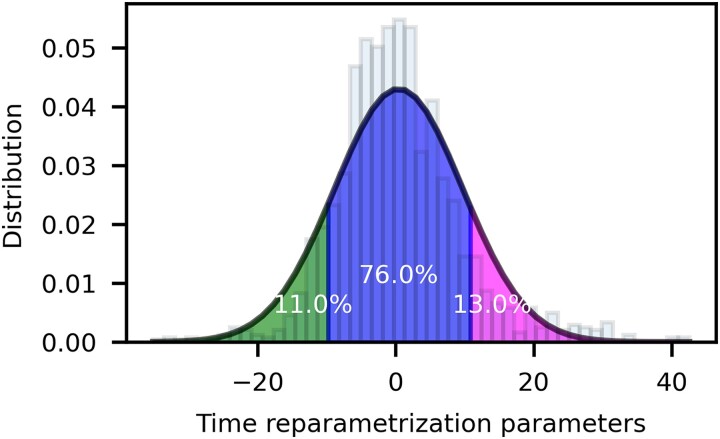
**Distribution of the estimated values of time reparameterization parameters on the training set.** On the lift the part of the distribution below 1 SD, corresponding to the 11% of participants identified as ‘slow progressors’; in the center the part of the distribution above 1 SD, corresponding to the 13% of participants identified as ‘fast progressors’; on the right the part of the distribution lying within 1 SD, corresponding to the 76% of participants identified as ‘normative progressors’.

Figure 2 shows the distribution of the time reparametrization parameters (the difference between ODD and RDD) as estimated by the GPMM in the training set. Scatter plot of RDD versus ODD is shown in Supplementary Fig. S2. We identify the presence of two outlier groups (in magenta and green in Fig. 2) with respect to the normative progression population (in blue in Fig. 2). Specifically, 167 participants (11%) were classified as slow progressors (G1), a sub-group of 193 participants (13%) were classified as fast progressors (G3), while the remaining 1161 participants (76%) were classified as normative progressors (G2).

Table 1 shows the heterogeneity in clinical presentation among the two outlier groups and the normative population. No differences were observed among the groups in terms of the rate of relapses or in the proportion of treated participants during the follow-up period. We note that fast progressors (G3) as compared to slow progressors (G1) are older at symptom onset (mean: 35.0 years versus 38.5 years, *P* < 0.001) and are males in a higher proportion (61.1% versus 48.5%, *P* = 0.013).

### Prognostic differences between fast and slow progressors

**Figure 3:**
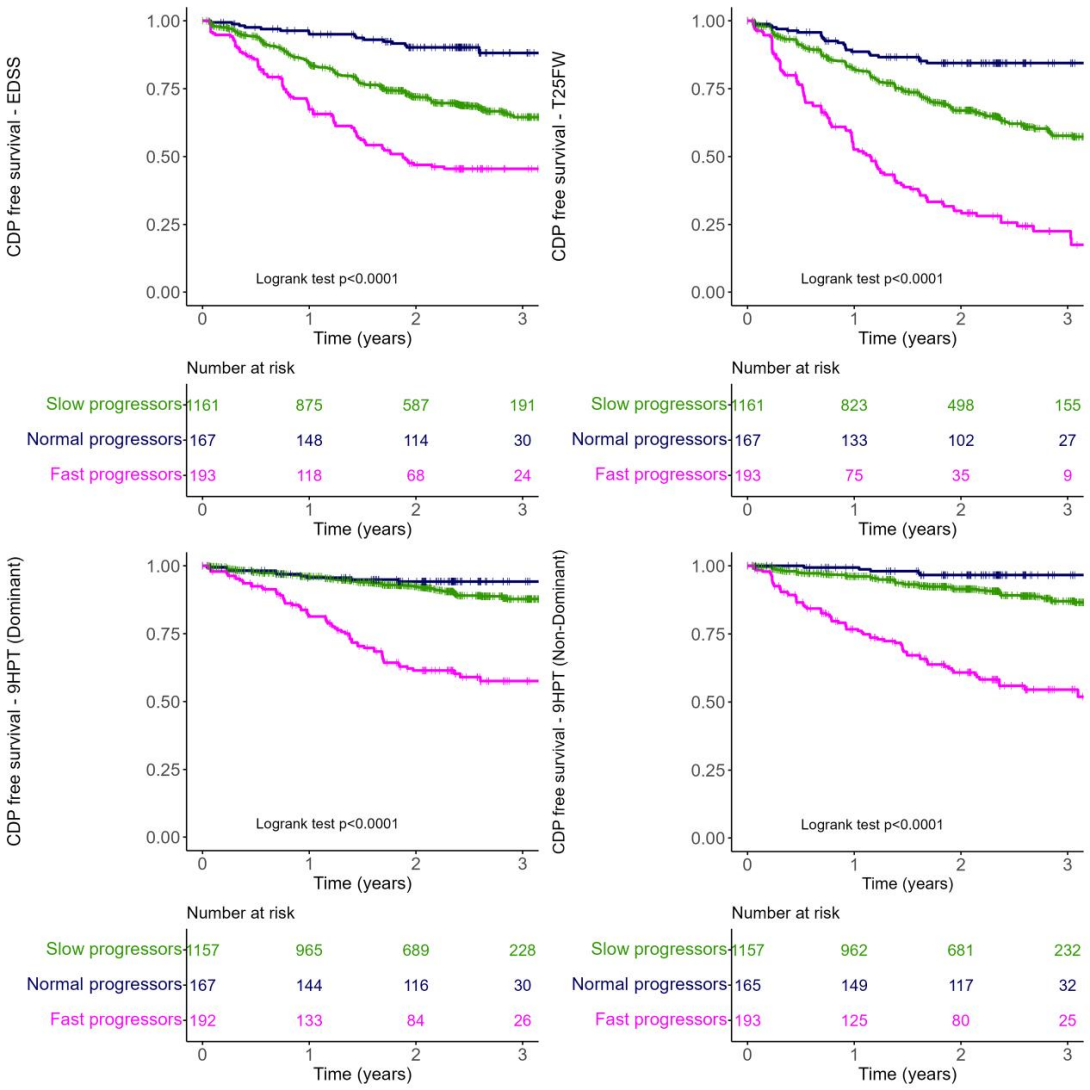
**Three-month confirmed disease progression on the PPMS sub-groups as identified by the model on the training set.** CDP computed on EDSS (*top*-left), T25FW (*top*-right), 9HPT dominant (*bottom*-left) and non-dominant (*bottom*-right) on the training set. Kaplan–Meier curves and the log-rank test were used to compare progression free survival distributions among the study groups.

Table 2 and Fig. 3 show a statistically significant difference in the rate of CDP computed on EDSS, T25FW, SDMT and 9HPT (both dominant and non-dominant), among the three populations defined by the GPPM (log-rank test for three-group comparison, *P* < 0.05 for all the disease activity measures). In particular, participants in the slow progressors group faced a 71% reduced risk of experiencing 3-month CDP as determined through EDSS when compared to those in the normative-progressor group (HR = 0.29, 95% CI: 0.17–0.47, *P* < 0.0001). Conversely, when we compared the fast progressors group to the normative-progressors group, the risk doubled (HR = 2.09, 95% CI: 1.66–2.62, *P* < 0.0001). Risk of CDP computed on T25FW and 9HPT is reduced by more than half when comparing slow progressors to normative-progressor individuals and, on the other hand, the risk increased by more than 3-fold comparing fast progressors to normative progressors (see Table 2). Only 42 CDP events were observed using SDMT as study outcome, with 3 occurring in the slow progressors and only 1 in the fast progressors. Such limited data precluded statistically robust comparisons between the groups.

**Table 2:** Cox model for 3-month confirmed disease progression computed on EDSS T25FW, and on 9HPT (dominant and non-dominant hand), stratified by the sub-group defined by GPMM in the training set (ARPEGGIO, OLYMPUS and ORATORIO trials) and in the testing set (SPI2 trial)

	Training dataset (*N* = 1521)				Testing dataset (*N* = 227)			
	Univariable		Multivariable^a^		Univariable		Multivariable^a^	
	HR (95% CI)	*P*-value	HR (95% CI)	*P*-value	HR (95% CI)	*P*-value	HR (95% CI)	*P*-value
EDSS								
Slow versus normative progressors	0.29 (0.17–0.47)	<0.0001	0.29 (0.17–0.47)	<0.0001	0.18 (0.02–1.28)	0.087	0.21 (0.03–1.61)	0.134
Fast versus normative progressors	2.09 (1.66–2.62)	<0.0001	2.09 (1.66–2.63)	<0.0001	1.50 (0.76–2.95)	0.243	1.45 (0.73–2.88)	0.282
T25FW								
Slow versus normative progressors	0.39 (0.26–0.59)	<0.0001	0.38 (0.25–0.57)	<0.0001	0.35 (0.08–1.46)	0.110	0.43 (0.10–1.85)	0.257
Fast versus normative progressors	3.00 (2.43–3.69)	<0.0001	2.96 (2.40–3.65)	<0.0001	2.70 (1.47–4.97)	0.001	2.80 (1.50–5.24)	0.001
9HPT dominant								
Slow versus normative progressors	0.60 (0.30–1.18)	0.140	0.59 (0.29–1.16)	0.127				
Fast versus normative progressors	4.85 (3.55–6.62)	<0.0001	4.66 (3.41–6.37)	<0.0001				
9HPT non-dominant								
Slow versus normative progressors	0.31 (0.13–0.77)	0.011	0.29 (0.12–0.71)	0.007				
Fast versus normative progressors	5.32 (3.94–7.17)	<0.0001	5.15 (3.81–6.95)	<0.0001				

EDSS, expanded disability status scale; T25FW, Timed 25-Foot Walk test; 9HPT, 9-Hole Peg Test; SDMT, Symbol Digit Modalities test.

9HPT data were not available for the SPI12 trial; the SDMT data is available only in ARPEGGIO trial; only 3 CDP events observed using SDMT occurred in the slow-progressors and only 1 CDP event in the fast progressors; no CDP events computed based on SDMT were observed in SPI12 trial.

^a^Age at onset, treatment arm and sex were entered in the model.

To assess the potential for interactions between treatment and the sub-groups of participants defined by the GPPM on 3-month CDP, a sensitivity analysis was utilized with a treatment-by-sub-group interaction term as a covariate within the Cox regression model. Notably, this interaction term did not demonstrate statistical significance for any of the outcomes evaluated, even when the ARPEGGIO trial (the only trial without anti-CD20 drugs) was excluded (data not shown).

When classified in three populations using solely the ODD (the ‘null model’ described in ‘Statistical Analysis’), 263 (17.29%) of the participants were classified as slow progressors, 1021 (67.13%) as normative progressors and the remaining 237 (15.58%) as fast progressors. Differences in the rate of CDP calculated for the study outcomes among the three ‘null’ populations were not found (Supplementary Table S2).

The median age at EDSS 6 was 53.5 years (IQR: 48.0–56.5 years) in G1 participants, 49.0 years (IQR: 43.0–53.0 years) in G2 participants, and 44.0 years (IQR: 38–51 years) in the G3 sub-group. Supplementary Fig. S3 shows that the median time from the onset of disease symptoms to reaching a confirmed EDSS 6 was 32.78 years (95% CI: 30.08–35.48 years), 11.62 years (95% CI: 10.95–12.29 years) and 6.75 years (95% CI: 5.83–7.68 years) for G1, G2 and G3, respectively (log-rank test for three-group comparison *P* < 0.0001).

### Model testing in the external independent dataset

A total of 227 PPMS participants from the SPI2 trial were used as an external testing set and their demographic and clinical characteristics are detailed in Supplementary Table S1. The relapse rate and T1 lesion volume were similar between the training and the external testing datasets, while the population in SPI2 trial appeared to be slightly older and with a longer disease duration than the training set.

The predicted RDD of the testing individuals was computed, based on the trained model, estimating a probabilistic model for the individual temporal staging and computing its expectation.^17^ Then, following what was done on the training set, the normative and outlier sub-groups of progression were identified on the test set by examining the difference between individual ODD and model’s RDD. 25/227 (11.01%) late slow progressors (G1); 37/227 (16.30%) fast progressors (G3); and 165/227 (72.69%) normative progressors (G2) were identified.

Table 2 shows the difference in rate of 3-month CDP computed on EDSS, T25FW and SDMT, among the three test set sub-groups identified by the model. The results were all consistent with those found on the training set, even if the statistical significance was not reached, likely because of the small size of the test sample. Disease duration, EDSS, T25FW and SDMT (all at baseline) are significantly different in the sub-groups. Further, the slow progressors face a reduced risk of experiencing 3-month CDP determined through EDSS (HR = 0.21), while fast progressors have an increased risk (HR = 1.45). Similar effect sizes were obtained when determining the 3-month CDP through T25FW.

## Discussion

In this study, we identified the group-wise, multimodal longitudinal evolution timeline of disability, cognition and neurodegeneration in PPMS along an estimated timeline of roughly 50 years. To do so, we employed GPPM, a probabilistic data-driven disease progression model, on a large dataset encompassing longitudinal multimodal disability assessments (walking ability, upper limb function and cognitive function) and MRI measurements (lesion and brain volume).

The outcomes of our investigation revealed compelling insights, compatible with previous findings in longitudinal studies in PPMS. First, the EDSS reaches its highest rate of change at around 15 years along the estimated progression, plateauing after ∼30 years of disease duration. This suggests that the utility of EDSS diminishes in monitoring disease progression beyond this temporal threshold, advocating for the research of potentially superior alternative measures in later stages. Second, the 9-Hole Peg Test performed with the dominant hand displayed less variability compared to the non-dominant hand in the population, underscoring the significance of considering hand dominance in evaluating disease-related functional changes.^37^ NBV emerged as the metric with the slowest progression to its peak rate of change along the disease trajectory. To elucidate this phenomenon, it is crucial to examine NBV in relation to clinical deficits. Notably, tissue damage extend beyond primary lesions, encompassing injuries and damage in seemingly normal white matter (intralesional).^38^ This observation underscores the intricate interplay between structural changes and functional impairments. Furthermore, NBV emerges as a shared final pathway implicated in diverse processes, including the secondary damage associated with lesions.^39^ Finally, our model suggests that, although the increase in lesion volume over time is highly variable within the population, it remains relatively constant overall. This finding is consistent with studies on PPMS which also show a relatively constant increase in lesion volume over time, particularly during the early stages of the disease.^40^ These considerations highlight the multifaceted nature of neurodegenerative mechanisms and underscores the necessity for further investigation into the temporal dynamics of brain volume alterations correlated with clinical manifestations.

Our model allowed the data-driven identification of two subsets of outliers, representing individuals with either fast or slow progressions relative to the normative trajectory. Slow progressors can be interpreted as those individuals who, despite having a long disease duration, exhibit a level of disability comparable to those with a shorter disease duration—in other words, they appear to be performing better than normative progressors with the same disease duration. Conversely, fast progressors are participants who, despite a short disease duration, display a level of disability akin to those with a longer disease duration, as the GPPM aligns them accordingly. This observation tentatively suggests the presence of disease processes in these sub-groups that evolve at rates differing from the normative population. However, more targeted studies are required to elucidate the underlying pathophysiological mechanisms in these groups.

Distinctive characteristics were observed between these sub-groups in terms of baseline (defined as the time of enrolment in the trial) clinical and demographic features. Fast progressors (which have a shorted disease duration) were characterized by an older age at symptom onset and a higher male sex as compared to slow progressors. This statement is consistent with a prior study on PPMS participants,^10^ indicating a tendency for a higher prevalence of males and older individuals at symptom onset among those with more severe disability. Prognostically, the two sub-groups demonstrated divergent outcomes based on the 3-month CDP evaluated on EDSS, with fast progressors exhibiting a significantly worse prognosis.

Despite identifying two subsets of outliers based on their progression rate with respect to the normative population, Fig. 1 shows considerable variance in the estimated GPPM trajectories/sequence, suggesting potential existence of multiple sub-groups within the overall population, each potentially following distinct (sub)group-level trajectories. This observation suggests two key points: (i) the primary clinical utility of the current GPMM version lies in group-level applications such as clinical trials rather than individual monitoring and (ii) exploring these subtypes using a clustering-based version of GPPM could be a promising direction for future research^13,41^ (for clustering-based extensions of different disease progression models).

Machine learning models were previously applied to MS clinical trial data, to identify sub-groups of patients with different prognosis or different propensity to respond to treatment. The first paper applied an unsupervised machine learning algorithm, called Subtype and Staging Inference (SuStaIn),^29^ to uncover data-driven assessment of the pathological changes visible on a large pooled dataset of MRI scans. They showed that it is possible to identify three data-driven MS subtypes that do not coincide with traditional MS classifications (relapsing remitting, primary progressive, etc.) and that are much more strongly predictive of response to a particular treatment. While most of the clinical trials in PMS failed to detect any relevant treatment effect in the whole populations enrolled in these trials, the ‘lesion-led’ subtype showed a significant treatment response in the three trials in PMS (*n* = 2900) included in the analysis as the test set. However, another study observed that although the lesion-led subtype was associated with increased inflammatory activity and correlated with disease severity and functional impairment at baseline, this subtype did not reliably predict disability progression nor effectively differentiate treatment response heterogeneity.^42^ Conversely, the sub-groups delineated using GPPM displayed divergent outcomes based on 3-month CDP, yet they were unable to discern treatment effects. This finding aligns with individual clinical trial results, where the treatment effect estimated on clinical endpoints in the PPMS population was either absent or weak at best. These observations suggest that the molecular mechanisms targeted by the specific drugs in these clinical trials may have limited impact on disease progression rates, which is the case for Laquinimod (ARPEGGIO trial^31^). When we removed the ARPEGGIO trial and repeated the analysis we still observed no significant interaction for Rituximab and Ocrelizumab (ORATORIO and OLYMPUS trials). This indicates that while GPPM may not predict treatment response when it does not impact disease progression rate, it remains a valuable tool for understanding disease progression itself.

A novel deep learning framework was recently used to estimate an individual’s treatment effect on disability progression using readily available clinical information (demographic characteristics and clinical disability scores) and scalar MRI metrics (lesional and volumetric) collected in clinical trials.^43^ An intriguing result was that in this experiment a non-linear model (multi-layers perceptron) outperformed other linear (and log-linear) combinations of baseline variables, suggesting that complex relationships exist between the baseline features and treatment effect. Recently, an analysis using Bayesian deep learning was used for estimating the posterior distribution over factual and counterfactual outcomes on several treatments.^44^ The model was trained and tested to predict future new and enlarging T2 lesion counts on the Progressive MS Alliance dataset.

The present study has important limitations, including the relatively small size of the test set that contributed to the lack of statistical significance in testing on the external dataset. Addressing this limitation requires examining more extensive clinical trial data. One limitation of our model is the enforced global monotonicity of population-level trajectories to ensure identifiability. While this allows for small fluctuations within the whole timeline, it may not fully capture the non-monotonic nature of disease progression, even in PPMS, where progression is generally considered monotonic. PPMS patients can still experience significant fluctuations in disease activity, and enforcing global monotonicity may oversimplify these dynamics, potentially affecting the model’s accuracy. Future work should explore more flexible trajectory assumptions while maintaining identifiability to better reflect the heterogeneity and variability of disease progression.^18^ Another significant limitation is the use of the same markers (notably, the EDSS) for computing both the RDD and subsequent a posteriori statistical tests (CDP). This dual use introduces potential circularity, where EDSS scores used for initial group categorization may bias outcomes assessment. While ensuring internal consistency, this approach limits generalizability and robustness. Furthermore, the limitations of the EDSS are well known, particularly its reduced sensitivity to the clinical accrual of non-motor disability in subjects with progressive forms of the disease, compared to newer scales like the MS Functional Composite.^45^ However, we focused on the EDSS here because it remains the most widely used metric of overall disability in clinical trials. Future research, including data from broader datasets, will explore alternative metrics for a posteriori statistical testing to mitigate these limitations and enhance the study’s reliability.

Another limitation of this study is the use of +/− 1 SD as a cutoff point to define fast and slow progressor groups. We note that GPPM models the distribution of the time reparametrization parameters as Gaussian, simplifying the identification of the tails of the distribution by means of SDs or quantiles and obviating the need for outlier detection techniques (e.g. clustering). Nonetheless, it is possible to seek an optimized threshold by, for instance, maximizing the separation between the ‘outlier’ populations and the normative one. This would, however, introduce a bias on the analysis of such differences, and thus, we have not explored this strategy. Other ‘hard’ cutoff values (1.5 and 2 SD) were explored, and the results remained consistent, although the sample size was too small to draw definitive conclusions (see Supplementary Table S3). It is crucial to note that further research with larger samples may be necessary to confirm these findings and establish more robust criteria for defining progression groups.

Another noteworthy aspect for consideration pertains to the absence of ‘advanced’ metrics, such as MRI metrics related to the smoldering disease,^46,47^ chronic inflammation,^48^ regional brain and spinal cord atrophy^49^ and emerging fluid biomarkers.^50^ The rationale behind this omission stems from the inherent challenges associated with quantifying these parameters in randomized clinical trials. Despite these limitations, it is fundamental to acknowledge that the exclusion of these advanced measures may impact the comprehensive understanding of disease dynamics. Future research may benefit from employing innovative methodologies or collaborative frameworks to overcome these challenges and incorporate these critical metrics, thus contributing to a more nuanced and comprehensive understanding of disease progression.

In summary, the group-wise, multimodal longitudinal evolution timeline of disability, cognition and neurodegeneration in PPMS was described along an estimated timeline of roughly 50 years leading to identification of two sets of outliers experiencing either hastened or slowed progressions with respect to the normative population.

## Supplementary Material

fcae434_Supplementary_Data

## Data Availability

Data used in this study were collected within the International Progressive MS Alliance project (IPMSA, award reference number PA-1603-08175). Access requests should be forwarded to the relevant data controllers. SPI2 study data were made available by the SPI-2 investigators and are available upon request to qualified researchers. Source code for the GPPM algorithm is freely available at https://gitlab.inria.fr/epione/GP_progression_model_V2.git. This repository does not contain dataset-specific code, since the data we used are not publicly available.
