# Supplementary material for: A data-driven model of disability progression in progressive multiple sclerosis
Source: Brain Commun. 2024 Dec 3;7(1):fcae434. doi: 10.1093/braincomms/fcae434 (PMC11704797; doi:10.1093/braincomms/fcae434)
Supplement: fcae434_Supplementary_Data [file fcae434_supplementary_data.docx]

**
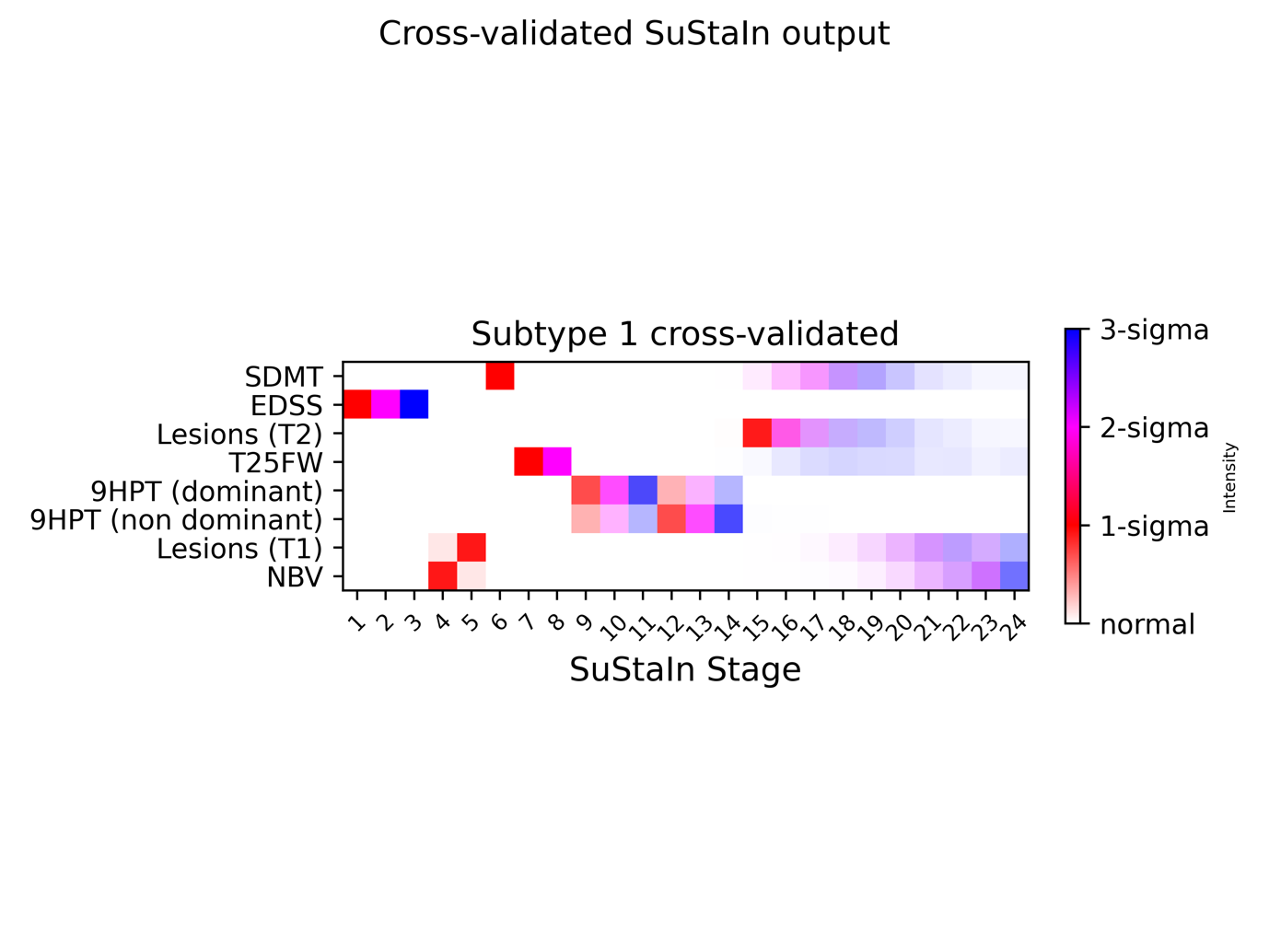
**

**Supplementary Figure 1.** Zscore-SuStain estimated sequence of events (cross-validated). The x-axis of the positional variance diagram represents the estimated stages along which the discrete model is indexed. Each stage corresponds to a disability assessment or MRI metric reaching a new z-score threshold from the set of z-scores for each feature, which is set to 3. Each entry of the positional variance diagram represents the probability that each feature has reached a specific z-score at that SuStaIn stage. Here, z-scores of 1 are shown in red, 2 in magenta and 3 in blue. SuStaIn uses MCMC sampling to estimate the uncertainty in the subtype progression patterns, here represented as the faded red, magenta or blue colours. The sequence is based on a cross-sectional dataset of 1521 individuals derived from the training set, where, for each individual, the time point with the largest number of features was selected. In cases of a tie, the earlier time point was chosen. The model implementation specifics include one progression type, 25 starting points, 10^5 MCMC samples, and 10-fold cross-validation.

**
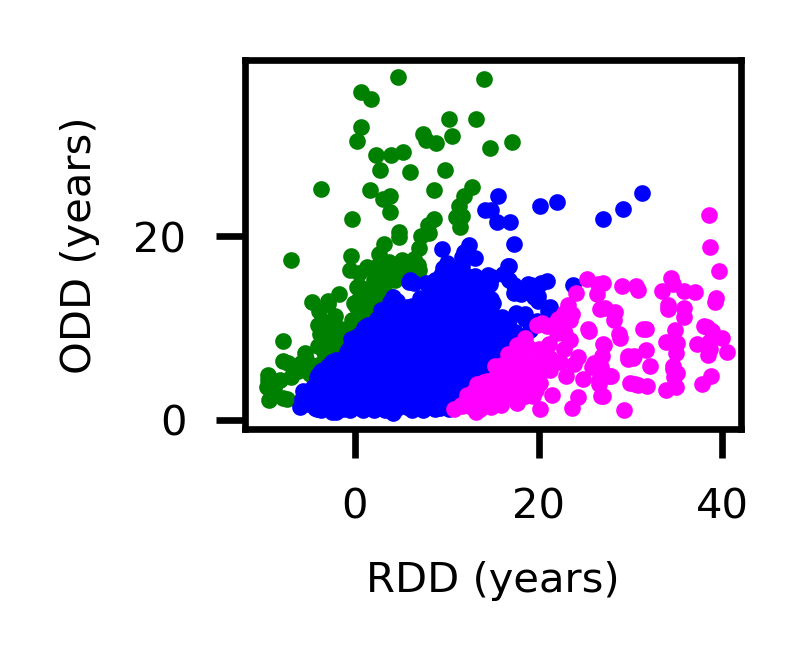
**

**Supplementary Figure 2**. Scatter plot of individual Reparametrised Disease Duration (x axis) VS Observed Disease Duration (y axis), coloured by GPPM-defined subgroups: blue = normative progressors; green = slow progressors; magenta = fast progressors. The plot includes the entire training dataset of 1521 individuals, with each dot representing one individual.

**
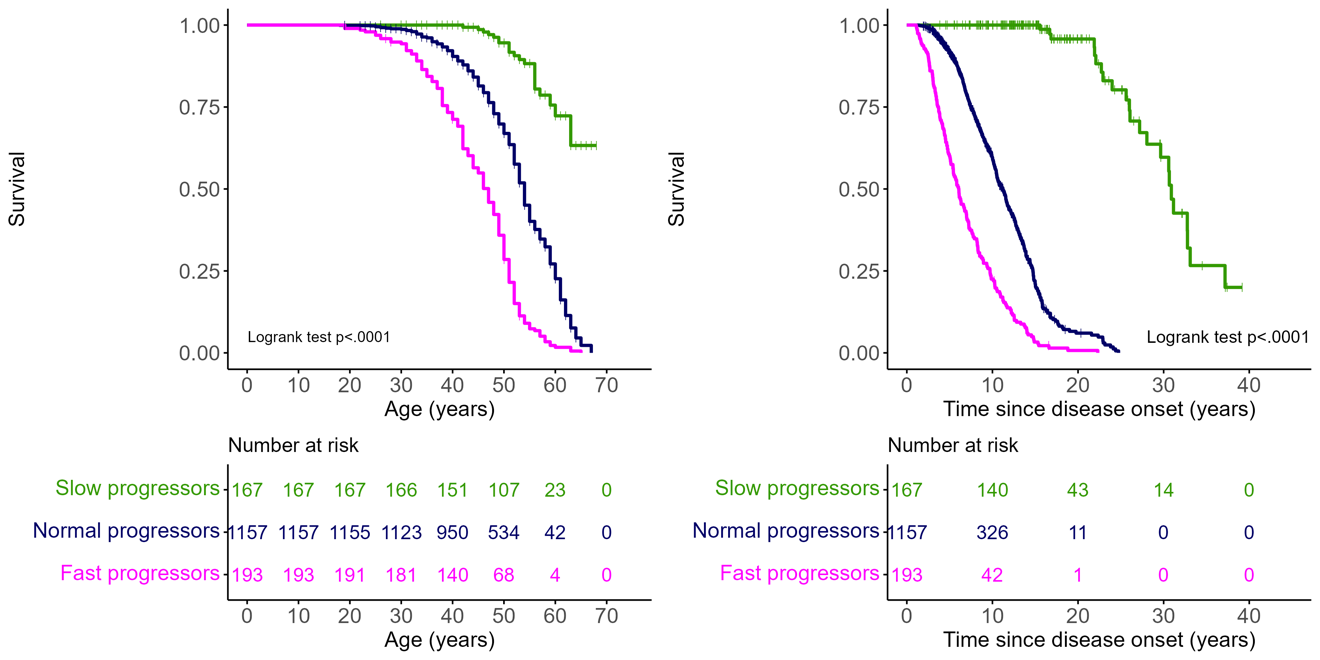
**

**Supplementary Figure 3.** Kaplan–Meier estimate of age to reach EDSS 6 (left) and of time from symptoms onset to EDSS 6 (right) according to the PPMS subgroups as identified by the model on the training set. The log-rank test were used to compare the survival distributions among the study groups.

|  | **Overall population** | **Slow Progressors** | **Normative Progressors** | **Fast Progressors** | **p-value** |
| --- | --- | --- | --- | --- | --- |
| **N** | 227 | 25 (11.01) | 165 (72.69) | 37 (16.30) |  |
| **Age at symptoms onset (years), mean (SD)** | 43.06 (9.22) | 35.86 (10.25) | 43.58 (8.83) | 45.64 (8.04) | 0.0007 |
| **Male sex, n (%)** | 119 (52.42) | 16 (64.00) | 81 (49.09) | 22 (59.46) | 0.245 |
| **Disease duration at baseline, median (IQR)** | 8.92 (5.61-14.81) | 17.95 (10.07-20.83) | 8.95 (5.61-13.29) | 7.28 (3.90-9.28) | <.0001 |
| **EDSS at baseline, median (IQR)** | 6.00 (4.50-6.00)  N=224 | 4.00 (3.50-4.00) | 5.50 (4.50-6.00)  N=163 | 6.50 (6.00-6.50)  N=36 | <.0001 |
| **T25FW at baseline, median (IQR)** | 8.80 (6.45-12.25)  N=225 | 6.05 (4.95-6.45) | 8.20 (6.65-11.00)  N=163 | 21.70 (14.40-30.40) | <.0001 |
| **SDMT at baseline, median (IQR)^#^** | 41.00 (33.00-49.00) | 46.00 (39.00-52.00) | 41.00 (33.00-49.00)  N=304 | 37.00 (28.00-46.00) | 0.021 |
| **NBV (ml) at baseline, mean (SD)** | 1402.66 (70.43)  N=226 | 1428.73 (168.73) | 1402.62 (71.42)  N=164 | 1385.21 (63.06) | 0.111 |
| **T2 lesion volume (ml), median (IQR)** | 12.78 (4.83-25.92)  N=226 | 8.51 (2.27-13.86) | 13.92 (5.60-26.06)  N=164 | 15.04 (6.01-28.05) | 0.042 |
| **T1 lesion volume (ml), median (IQR)^##^** | 1.79 (0.47-3.91)  N=226 | 1.31 (0.04-2.62) | 1.78 (0.48-4.34)  N=164 | 2.43 (1.14-4.12) | 0.079 |
| **ARR during follow-up, mean (SD)** | 0.06 (0.20) | 0.09 (0.21) | 0.06 (0.20) | 0.05 (0.15) | 0.565 |
| **Relapse during follow-up, n (%)** |  |  |  |  | 0.770 |
| **0** | 204 (89.87) | 21 (84.00) | 150 (90.91) | 33 (89.19) |  |
| **1** | 22 (9.69) | 4 (16.00) | 14 (8.48) | 4 (10.81) |  |
| **>1** | 1 (0.44) | 0 (0.00) | 1 (0.61) | 0 (0.00) |  |
| **Treated patients, n(%)** | 117 (51.54) | 9 (36.00) | 92 (55.76) | 16 (43.24) | 0.100 |

**Supplementary Table 1.** Demographic and clinical characteristics of the three subgroups defined by GPPM in the testing dataset (SPI2 clinical trial). SD: standard deviation; EDSS: Expanded Disability Status Scale; IQR: interquartile range; T25FW: timed 25-foot walk test; SDMT: symbol digit modalities test; NBV: normalized brain volume; ARR: annualized relapse rate.

|  | **Training dataset (ARPEGGIO, OLYMPUS, ORATORIO)**  **N=1521** | | | |
| --- | --- | --- | --- | --- |
|  | **univariable** | | **multivariable*** | |
|  | **HR (95%CI)** | **p-value** | **HR (95%CI)** | **p-value** |
| **EDSS** |  | | | |
| slow vs normative | 0.89 (0.69-1.15) | 0.360 | 0.88 (0.68-1.13) | 0.315 |
| fast vs normative | 0.92 (0.70-1.20) | 0.533 | 0.94 (0.71-1.24) | 0.647 |
| **T25FW** |  | | | |
| slow vs normative | 1.00 (0.80-1.25) | 0.997 | 1.02 (0.81-1.29) | 0.838 |
| fast vs normative | 0.86 (0.66-1.12) | 0.265 | 0.83 (0.64-1.09) | 0.184 |
| **9HPT dominant** |  |  |  |  |
| slow vs normative | 0.84 (0.56-1.26) | 0.399 | 0.83 (0.55-1.26) | 0.391 |
| fast vs normative | 0.85 (0.54-1.32) | 0.469 | 0.83 (0.52-1.30) | 0.414 |
| **9HPT non dominant** |  | | | |
| slow vs normative | 1.05 (0.72-1.53) | 0.781 | 1.10 (0.76-1.61) | 0.612 |
| fast vs normative | 0.81 (0.51-1.27) | 0.351 | 0.73 (0.46-1.16) | 0.179 |

**Supplementary Table 2.** Cox model for 3-month confirmed disease progression computed on EDSS T25FW, and on 9HPT (dominant and non-dominant hand), stratified by the subgroups obtained using the criteria of mean +/-1 SD of the observed disease duration (i.e. the null model). *age at onset, treatment arm and sex were entered in the model. 9HPT data were not available for the MS-SPI trial.

|  | **Training dataset (ARPEGGIO, OLYMPUS, ORATORIO)**  **N=1521** | | | |
| --- | --- | --- | --- | --- |
|  | **univariable** | | **multivariable*** | |
|  | **HR (95%CI)** | **p-value** | **HR (95%CI)** | **p-value** |
| **EDSS** |  | | | |
| slow vs normative | 0.58 (0.22-1.55) | 0.279 | 0.63 (0.23-1.71) | 0.403 |
| fast vs normative | 2.24 (1.59-3.15) | <.0001 | 2.27 (1.61-3.22) | <.0001 |
| **T25FW** |  | | | |
| slow vs normative | 0.38 (0.12-1.17) | 0.091 | 0.36 (0.11-1.13) | 0.081 |
| fast vs normative | 3.17 (2.33-4.31) | <.0001 | 3.09 (2.25-4.25) | <.0001 |
| **9HPT dominant** |  |  |  |  |
| slow vs normative | - | - | - | - |
| fast vs normative | 6.23 (4.24-9.15) | <.0001 | 5.66 (3.79-8.44) | <.0001 |
| **9HPT non dominant** |  | | | |
| slow vs normative | 0.46 (0.06-3.27) | 0.436 | 0.39 (0.05-2.85) | 0.354 |
| fast vs normative | 6.27 (4.29-9.15) | <.0001 | 5.72 (3.87-8.48) | <.0001 |

**Supplementary Table 3.** Cox model for 3-month confirmed disease progression computed on EDSS T25FW, and on 9HPT (dominant and non-dominant hand), stratified by the subgroups defined by GPMM (+/-1.5 SD). Slow (N=25); Normative (N=1426); Fast (N=70). *age at onset, treatment arm and sex were entered in the model
